# Brain patterning perturbations following PTEN loss

**DOI:** 10.3389/fnmol.2014.00035

**Published:** 2014-05-14

**Authors:** Biliana O. Veleva-Rotse, Anthony P. Barnes

**Affiliations:** ^1^Neuroscience Graduate Program, Oregon Health and Science UniversityPortland, OR, USA; ^2^Department of Pediatrics, Oregon Health and Science UniversityPortland, OR, USA; ^3^Department of Cell and Developmental Biology, Oregon Health and Science UniversityPortland, OR, USA

**Keywords:** PTEN phosphohydrolase, brain development, mouse models, signal transduction, progenitor cells, axon outgrowth

## Abstract

This review will consider the impact of compromised PTEN signaling in brain patterning. We approach understanding the contribution of PTEN to nervous system development by surveying the findings from the numerous genetic loss-of-function models that have been generated as well as other forms of PTEN inactivation. By exploring the developmental programs influenced by this central transduction molecule, we can begin to understand the molecular mechanisms that shape the developing brain. A wealth of data indicates that PTEN plays critical roles in a variety of stages during brain development. Many of them are considered here including: stem cell proliferation, fate determination, polarity, migration, process outgrowth, myelination and somatic hypertrophy. In many of these contexts, it is clear that PTEN phosphatase activity contributes to the observed effects of genetic deletion or depletion, however recent studies have also ascribed non-catalytic functions to PTEN in regulating cell function. We also explore the potential impact this alternative pool of PTEN may have on the developing brain. Together, these elements begin to form a clearer picture of how PTEN contributes to the emergence of brain structure and binds form and function in the nervous system.

## Introduction

**Figure 1:**
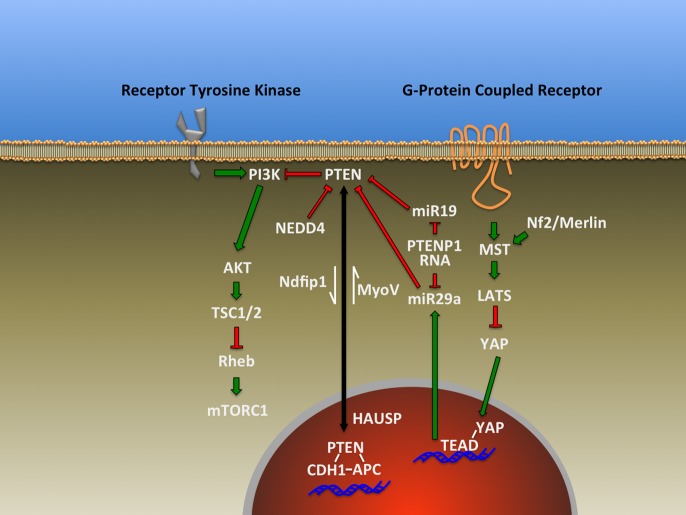
**Representative signal transduction pathways that have been found to either influencing or impacted by PTEN as described in the text**.

**Figure 2:**
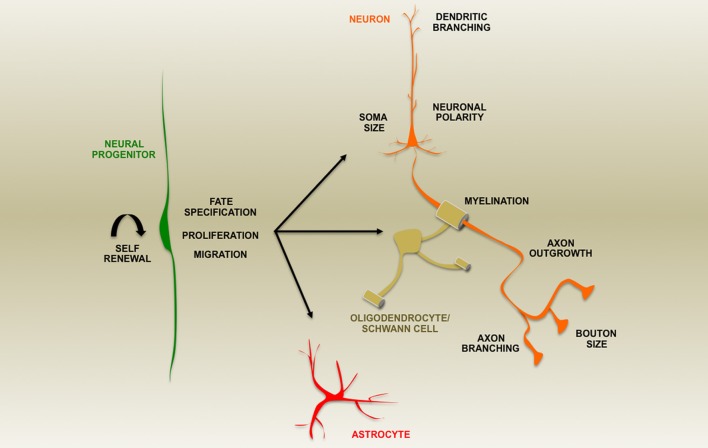
**A summary of aspects of nervous system development altered by loss of PTEN expression during development or in post-natal ablation studies as indicated by the text**.

Phosphatase and TENsin homolog (PTEN) is a protein/lipid phosphatase that is responsible for regulating a myriad of signal transduction events in the brain including those essential for central nervous system patterning. It is an ancient component of many highly conserved signaling pathways including most notably the Phosphatidyl Inositide 3-Kinase (PI3K)/mammalian Target Of Rapamycin Complex 1 (mTORC1) pathway for which PTEN serves as a major antagonist (Figure 1). This article will explore the roles of this enzyme based on findings made primarily using genetic loss-of-function studies within the developing and adult nervous system and disciplines beyond where appropriate. Recent reviews have addressed the general cell biological functions of PTEN as well as the regulatory mechanisms controlling its activity and expression (Song et al., 2012; Ortega-Molina and Serrano, 2013). Many of its roles in mature nervous system physiology and pathology are tackled by other articles within this *Research Topic*. Here, we explore diverse aspects of brain development influenced by PTEN including progenitor cell proliferation, cell fate determination, migration, polarity, axon-dendrite morphogenesis, cell soma size, and myelination (Figure 2). The observations discussed in this review reveal PTEN's key roles in cellular signaling that have the potential to be harnessed via cellular, pharmacologic and genetic strategies to improve disease outcomes (Yang et al., 2009; Ortega-Molina and Serrano, 2013; Maire et al., 2014).

## Cell proliferation/cell fate

The control of cell cycle timing and differentiation in the developing brain are essential elements for populating the nervous system with the appropriate numbers and types of cells. Multiple studies have demonstrated a key role for PTEN in stem cell maintenance and fate specification utilizing Cre recombinase mouse strains to conditionally knockout (cKO) PTEN expression in various populations of cells in the embryonic retina and brain, early post-natal, and adult brain regions including the cerebellum, cortex, hippocampus. We have included an accompanying table listing the Cre lines discussed in this review along with their primary reference and expression patterns (Table 1). Examination of regulated loss-of-function models has resulted in many unifying themes of PTEN function and observations that implicate extra-cellular, trans-cellular or cell-type specific roles for PTEN. While Cre recombinases offer a tremendous asset for understanding tissue and cell-type specific gene function, it is important to consider that recent work indicates that the timing of recombination may not correspond with the expected promoter-based expression pattern (Liang et al., 2012; Harno et al., 2013).

**Table 1:** **Summary of Cre lines cited in this publication**.

**Cre line**	**Publication cited herein**	**Original publication**	**Transgene or knockin**	**Construct details**	**Age: expression pattern**
CamKII alpha	Sperow et al., 2012	Tsien et al., 1996	Transgene	Alpha CamKII promoter-cre-pA; 11.1 kb	**P18 - Adult:** forebrain and hippocampus—CA1/CA3 and some dentate gyrus in hippocampus, some cortex, and striatum
Chx10	Sakagami et al., 2012	Rowan and Cepko, 2004	Targeted knock-in	Chx10 promoter-GFPcreIRESAP-FRT-Tn5/PGK promoter-Neo/Kan-FRT	**E11.5, P0:** outer neuroblastic layer of retina, nonpigmented prospective ciliary body; high expression; **P6:** ascending processes of bipolar cells; **P10:** cell bodies and developing dendrites of bipolar cells; **P23:** axons and dendrites of bipolar cells; mostly in inner nuclear layer
CNP1	Goebbels et al., 2006	Lappe-Siefke et al., 2003	Targeted knock-in	Exon 1 of CNP gene: ATG-Cre-PGK promoter-puromycin	**Adult:** cerebellar white matter; CNS white matter tracts
DAT	Inoue et al., 2013	Zhuang et al., 2005	Targeted knock-in	5′UTR of DAT-NLS-Cre-FRT-PGK promoter-Neo-polyA-FRT; crossed to FLP deleter line to remove PGK cassette	Specificity confirmed to dopaminergic neurons by tyrosine hydroxylase staining; seen in VTA and SNc
DAT	Diaz-Ruiz et al., 2009	Backman et al., 2006	Targeted knock-in	IRES-NLS-Cre-FRT-PGK promoter-Neo-polyA-FRT-3′UTR of DAT locus; crossed to FLP deleter line to remove PGK cassette	**E15, E17, P0:** VTA, substantia nigra, retrorubral field; low expression in glomeruli of olfactory bulb
Emx1	Lehtinen et al., 2011	Gorski et al., 2002	Targeted knock-in	IRES-Cre-intron/PolyA-PGK promoter-neo—3′UTR of emx1	**E10.5:** shows dorsal telencephalon as well as cranial nerve expression; **E12.5:** shows dorsal pallium and lateral pallium expression; **Adult brain:** mitral and deep periglomerular olfactory bulb, cingulate, cortex, hippocampus, endopiriform nucleus (ventral and dorsal), amygdaloid nucleus, nucleus of lateral olfactory tract, nuc of accessory olfactory tract, amygdalohippocampal area, basolateral amygdala, corpus callosum, anterior commisure, basomedial amygdala, posteromedial amygdaloid nucleus; **Summary:** progenitors and projection neurons and Cajal Retzius cells and oligodendrocytess from pallium as well as astrocytes express emx1 but inhibitory interneurons do not
En2 (Engrailed 2)	Marino et al., 2002	Zinyk et al., 1998	Transgene	En2 enhancer-En2 minimal promoter fragment-Cre cDNA	Embryonic dorsal mid-hindbrain junction starting as early as **E9.5** and staying consistent at **E14.5** and **adult**
GFAP	Kwon et al., 2001; Chalhoub et al., 2006	Kwon et al., 2001	Targeted knock-in	Disruption of GFAP exon1 by Cre insertion	**Postnatal** onset: Granule cells of cerebellum and dentate gyrus; very low expression in astrocytes and pyramidal cells
hGFAP	Fraser et al., 2004, 2008; Chalhoub et al., 2009	Bajenaru et al., 2002	Transgene	hGFAP promoter-Cre-IRES-Nuclear LacZ; 8 kb construct	**E11.5–E14.5:** brain first then spinal cord and optic nerves; **E18.5:** brain, optic nerves, and spinal cord; **P7:** astrocytes of hippocampus, cerebellum, ventral forebrain, and in Bergmann glia in cerebellum
hGFAP	Yue et al., 2005; Wen et al., 2013	Zhuo et al., 2001	Transgene	5′ flanking region of hGFAP promoter-nuclear Cre-mous protamine gene intron and polyA; 2.2 kb construct	**E13.5:** dorsal and medial telencephalon, with full neuraxis expression by birth; **5 weeks:** high expression in cortex, hippocampus, cerebellum, low expression in midbrain; also low expression in liver (ductal cells); expression in Bergmann glia of cerebellum as well as in granule layer
mGFAP	Gregorian et al., 2009	Gregorian et al., 2009	Transgene	Full murine GFAP gene sequence-Cre; 15 kb construct	**Adult** neural stem/progenitor cells—seen in supependymal zone (SEZ), rostral migratory stream, olfactory bulb; cre-expressing cells have nestin and GFAP staining in SEZ
L7	Marino et al., 2002	Oberdick et al., 1990	Transgene	Full L7 gene with LacZ cDNA in 4th exon	Purkinje cells in the cerebellum; bipolar neurons in retina; low expression in interpeduncular nucleus
Nestin	Groszer et al., 2001	Zimmerman et al., 1994; Bates et al., 1999	Transgene	Rat nestin promoter-intron 2 enhancer-Cre	Expression beginning at 18 somite stage; **E9.5:** ventral neural tube, and spinal cord; **E10.5** most of CNS; **E12.5:** in brain and spinal cord; some expression in heart and lungs, skin, limbs adult: also some in salivary gland, kidney, olfactory bulb; **E18.5** - expressed in progenitors of brain
Nestin Cre-ERT2	Bonaguidi et al., 2011; Zhu et al., 2012	Balordi and Fishell, 2007	Transgene	Rat nestin promoter-intron 2 enhancer-tk promoter-Cre ERT2-SV40 PolyA	Typical nestin expression pattern—**E14.5:** neural progenitors; **P60:** very low spontaneous recombination—only in presence of tamoxifen does expression increase substantially; expression patterns as reported for nestin-cre in embryo and adult, and last up to 2 month after tamoxifen pulse
Nestin Cre-ERT2	Amiri et al., 2012	Li et al., 2008b	Transgene	Rat nestin promoter/enhancer-Cre ERT2 fusion protein cDNA-inverse Nestn 2nd intron	Expression in previously reported (see above) nestin patterns only after tamoxifen induction, with some background in thalamus
Nex	Kazdoba et al., 2012	Goebbels et al., 2006	Targeted knock-in	Nex coding region of exon2 replaced by Cre-sense oriented NeoR-end of exon 2	Only in pyramidal cells of cortex, **E12, E13.5, E16.5:** expressed in differentiating cells but not progenitors; **Adult:** in cortex and hippocampus, dorsal horn of spinal cord, periglomerular layer of olfactory bulb, mitral layer of olfactory bulb, habenular nuclei, amygdaloid nuclei, piriform cortex, external cuneate nucleus, lateralreticular nucleus, nucleus of solitary tract, spinal trigeminal nucleus; only seen in neurons *in vivo* and *vitro*; marks glutamatergic neurons in cortex, no interneurons
Nse	Kwon et al., 2006a,b; Zhou et al., 2009	Kwon et al., 2006b	Transgene	Nse promoter-NLS-Cre-SV40 PolyA; 3.5 kB construct	Layers 3–5 of cortex; CA3, dentate gyrus, polymorphic layer and outer granular layer of hippocampus; **E13.5:** spinal cord and PNS during development; **P0:** spinal cord, DRG, trigeminal ganglia; **4 weeks:** kidney and testes and stomach; in neurons but not glia; ~55% of cortex, ~40% CA3, ~50% dg, ~60% PML
Olig2	Harrington et al., 2010; Maire et al., 2014	Schuller et al., 2008	Targeted knock-in	TVA-IRES-Cre-NeoR-Olig2	**P7:** 98% of white matter cells express it; small subpopulation of CGNP's in EGL (cerebellar lobes 9 and 10); **P21:** fate mapping to GC's in IGL; few rRL cells can generate Pax6+ CGNPs of EGL and GC's in IGL; rostral rhombic lip progenitors, oligodendrocyte precursor cells, NG2 cells, interneurons
Pax6 alpha	Cantrup et al., 2012; Jo et al., 2012	Marquardt et al., 2001	Transgene	Alpha-P0 promoter-Cre-IRES-GFP-intron-polyA; 5.5 kb	**E10.5:** onset of expression in neural retina, granule cell layer, inner nuclear layer, outer nuclear layer
Plp1-ERT2	Goebbels et al., 2010; Snaidero et al., 2014	Leone et al., 2003	Transgene	15 kb of PLP gene regulatory region-Cre-ERT2	**8 weeks:** Schwann cells, major white matter structures in CNS only after Tamoxifen treatment

Abbreviations: CGNP, cerebellar granule neuron precursor; CNS, central nervous system; DRG, dorsal root ganglia; EGL, external granular layer; ERT2, estrogen receptor regulated by tamoxifen; GC, granule cell; IGL, inner granular layer; IRES, internal ribosomal entry site; LacZ, Beta galactosidase; NeoR, neomycin resistance; NG2, NG2 proteoglycan +; NLS, nuclear localization signal; PGK, mouse phosphoglycerate kinase 1; PNS, peripheral nervous system; PolyA, polyadenylation signal; SNc, substantia nigra pars compacta; TVA, avian tumor virus receptor A; VTA, ventral tegmental area.

### Developing retina/olfactory bulb

Conditional removal of PTEN expression in the developing retina in Paired box 6 (Pax6)-Cre or CEH10 Homeodomain Containing Homolog promoter-driven Cre (Chx10^Cre^) mice results in alterations in progenitor maintenance and specification/differentiation (Cantrup et al., 2012; Sakagami et al., 2012). Pax-6-Cre-mediated deletion results in accelerated neurogenesis and premature loss of retinal progenitor cells accompanied by tissue hypertrophy not attributed to excessive stem cell production (Cantrup et al., 2012). This is in contrast to observations from other neuronal progenitor populations (see below) and illustrates the importance of spatio-temporal context in determining the cellular consequence to PTEN loss. Interestingly, recent evidence also from the developing retina indicates that PTEN is necessary in Notch-coordinated neurogenesis by downregulating Akt/protein kinase B signaling and allowing the intracellular domain of Notch to form a transcriptionally active complex in the nucleus (Jo et al., 2012). In contrast, increased PTEN expression in the developing retina of mice lacking the transcriptional repressive orphan nuclear receptor TLX/NR2E1 has been proposed to contribute to decreased proliferation and increased apoptosis (Zhang et al., 2006). Similar progenitor cell alterations in the cerebral cortex of TLX-null mice are thought to be due to parallel perturbations in PTEN signaling as well as in expression of cyclins and cyclin regulatory proteins (Li et al., 2008a). Given the broad scope of genomic loci potentially targeted by the TLX repressor complex, more work remains to resolve the relationships between the phenotypes such as altered cell proliferation and apoptosis observed following loss of either PTEN or TLX/NR2E1. Olfactory bulb (OB) progenitor cells exhibit both similar and distinct responses to PTEN modulation. In this case, expression of a catalytically inactive form of PTEN led to increased differentiation, and over-expression of PTEN in OB progenitors was observed to inhibit differentiation without affecting cell survival or proliferation (Otaegi et al., 2006). Treatment with insulin-like growth factor 1 (IGF1) was able to overcome this suppression, reinforcing the importance of extra-cellular influences on PTEN's impact on transduction. This effect of insulin-like growth factor on progenitor proliferation is reinforced by a recent study exploring the genetic interaction of the apical complex protein Pals1 and PTEN (Lehtinen et al., 2011). This study showed that PTEN enhances signaling of insulin-like growth factors present within cerebrospinal fluid through regulation of the IGF1 receptor expression.

### Embryonic neural stem cells

Embryonic studies using various Cre recombinase deletion lines share the common feature that elimination of PTEN expression results in alterations in stem cell niche maintenance and proliferation (Backman et al., 2001; Groszer et al., 2001; Kwon et al., 2001; Fraser et al., 2004; Kwon et al., 2006a; Fraser et al., 2008; Gregorian et al., 2009). Similar observations have been made regarding adult neurogenesis in the hippocampus and sub-ependymal zone (SEZ)/sub-ventricular zone (SVZ) (Bonaguidi et al., 2011; Amiri et al., 2012; Zhu et al., 2012). The observations that PTEN loss accelerates G1/S transition (Sun et al., 1999) and promotes G0 exit into the cell cycle (Groszer et al., 2006) provide an excellent backdrop to consider the effects of PTEN deletion in neural progenitors. Given the early lethality of whole-body PTEN deletion, Nestin-Cre provided one of the first avenues into how neural progenitors respond to loss of this key regulatory molecule (Groszer et al., 2001). An increase in Bromodeoxyuridine (BrdU)-labeled cells in the telencephalic ventricular zone coupled with reduced TUNEL staining indicated a derangement in the normal balance of neural progenitors. Embryonic day 14.5 neurosphere cultures from PTEN mutant mice displayed an increase in proliferative capacity compared to controls. Postnatal analysis of mice expressing a version of glial fibrillary acidic protein (GFAP) promoter-directed Cre did not detect significant alterations in cell proliferation, but observed other phenotypes including seizures, ataxia, and macrocephaly (Backman et al., 2001; Kwon et al., 2001). Prenatal loss of PTEN, mediated by Human GFAP (hGFAP) promoter-regulated Cre, results in a significant increase in the number of astrocytes following deletion (Fraser et al., 2008). This difference in the effect of PTEN loss highlights cell-type specific responses and sensitivity to the timing of the Cre recombination deletion during development. Cre recombinase regulated by the dorsal telencephalon-restricted transcription factor Emx1 promoter also reveals an expanded progenitor proliferation following embryonic deletion of PTEN (Lehtinen et al., 2011).

Conditional cerebellar PTEN deletion using Engrailed 2 (En2)-Cre revealed decreased proliferation and cell death (Marino et al., 2002). Premature differentiation of Bergmann glia has been detected by studies focused on the cerebellum of floxed PTEN mice either using hGFAP-Cre, or mice injected with virally encoded Cre (Yue et al., 2005). Together, these studies place PTEN as a modulator of proliferative/apoptotic balance during embryonic brain development.

### Adult neural stem cells

Adult neurogenesis is believed to represent a continuation or recapitulation of embryonic programs in privileged cellular niches, namely the subgranular zone of the dentate gyrus and SEZ/SVZ. The role of PTEN in this progenitor population has been pursued using four distinct Cre mouse lines, including a third version of GFAP driven Cre (mGFAP) with expression limited to postnatal progenitors (Gregorian et al., 2009). The mGFAP line was used to delete PTEN and determine the effect in SEZ progenitors. Neurospheres derived from these animals demonstrated an enhanced ability for self-renewal (Gregorian et al., 2009), a result paralleling previous observations in neurospheres derived from embryonic tissue (Groszer et al., 2001, 2006).

The effect of PTEN loss in the SEZ/SVZ progenitors has also been explored using three estrogen receptor T2 (tamoxifen-inducible) Cre (ERT2-Cre) lines controlled by the Nestin promoter (Nestin-ERT2) (Bonaguidi et al., 2011; Amiri et al., 2012; Zhu et al., 2012) but no significant difference in proliferating progenitors of the SVZ was found (Zhu et al., 2012). However, an expanded SEZ/SVZ and rostral migratory stream (RMS) of cells migrating to the OBs was noted, similar to that observed using mGFAP-Cre (Gregorian et al., 2009) or Olig2^Cre^ (Maire et al., 2014) deletion of PTEN, both of which eliminate PTEN in the SVZ. The expansion in the case of the Nestin-ERT2 animals was found to be due, in part, to ectopically differentiated neurons in both locations as opposed to the typical neuroblasts found in these regions, consistent with perturbed differentiation following PTEN ablation. The SVZ expansion in this particular model was also shown to be dependent on mTORC1 signaling as rapamycin treatment was able to prevent it (Zhu et al., 2012). A role for PTEN in the adult neurogenic niche of the dentate gyrus where stem cells exist at various stages of lineage from neural stem cells to intermediate progenitors and finally nascent neuroblasts has also been explored using cre mouse strains. This cell population was also targeted by using Nestin-ERT2 Cre in which either single clonal cells (Bonaguidi et al., 2011) or ensembles of cells (Amiri et al., 2012) were analyzed. Bonaguidi et al. employed the Mosaic Analysis with Double Markers (MADM) to sparsely label progenitor cells and their progeny via Cre recombination of split fluorescent protein reporters (Zong et al., 2005). This approach permitted lineage tracing from particular progenitors to detect proliferation and differentiation of individual clonal populations. Three distinct observations were made from these clones one month after labeling and deletion of PTEN: (1) There was a reduction in quiescent progenitors, (2) clones had predominately undergone symmetric division-based stem cell expansion and (3) clones had depleted their progenitors and consisted of astrocytes, cells transitioning into astrocytes, or neurons (Bonaguidi et al., 2011). The majority of clones fell into the last category, indicating a failure to maintain stem cell identity by those progenitors that lacked PTEN expression. This parallels the findings of Amiri et al. that neurospheres derived from PTEN-null progenitors had an increased propensity to undergo differentiation following growth factor withdrawal (Amiri et al., 2012). Similarly, using BrdU to fate map the progeny following acute deletion of PTEN, it was revealed that progenitor depletion followed an early surge of neuro/glio-genesis, with a bias toward astrocyte generation similar to that observed by Bonaguidi et al.

The results discussed above indicate the importance of PTEN expression and emerging data from microRNA studies indicate that cells have mechanisms to post-transcriptionally regulate PTEN levels. Recent evidence implicating the miR-17-92 microRNA cluster in regulating neural progenitor expansion and differentiation also demonstrates that targeting PTEN expression is one mechanism involved via miR-19, a microRNA encoded within the miR-17-92 cluster (Figure 1) (Bian et al., 2013). Interestingly, an additional level of regulation may exist for PTEN based on the discovery that a processed pseudogene RNA, PTENP1, can act as a microRNA decoy or competing endogenous RNA (ceRNA) (Figure 1)(Salmena et al., 2011) to negate or modulate the effect of microRNAs on PTEN expression (Poliseno et al., 2010). Future studies will be required to define the developmental, cell-type and species-specific expression patterns for this pseudogene in the nervous system, as one study found that mice lack PTEN pseudogenes (Kwabi-Addo et al., 2000). Similarly, the transcriptional co-activator Yes-associated protein (YAP) has been shown to negatively regulate PTEN levels by controlling microRNA miR-29a transcription (Figure 1) (Tumaneng et al., 2012). Remarkably, over-expression of YAP and its co-activator TEAD, through up-regulation of cell cycle genes such as Cyclin D1 increases neural progenitor proliferation (Lavado et al., 2013). However, Cyclin D1 expression does not appear to account for the effect of YAP/TEAD. It is possible that miR-29a may mediate these effects by lowering PTEN levels, contributing to increased progenitor proliferation. Telencephalic deletion of the tumor suppressor Neurofibromatosis 2 (NF2)/Merlin increases transcription of YAP/TEAD targets (Figure 1), resulting in increased progenitor proliferation in the cortex and hippocampus (Lavado et al., 2013) similar to that seen in PTEN loss. This observation represents a tantalizing link between PTEN and NF2, two proteins previously described as tumor suppressors. These data regarding microRNAs, along with a wealth of data demonstrating that PTEN is regulated at both transcriptional and post-translation levels (Song et al., 2012; Ortega-Molina and Serrano, 2013), indicate the tremendous amount of cellular resources dedicated to regulating PTEN expression, localization and enzymatic activity. It is unknown what portion of the cell cycle/differentiation alterations following deletion of PTEN in the nervous system are the result of disregulated signal transduction related to the PI3K/mTORC1 pathway. Recent discoveries offer the possibility that additional, non-phosphatase dependent modes of action may exist for PTEN in the nucleus contributing to cell fate.

An emerging aspect of PTEN regulation of cell fate arises from the nuclear pool of PTEN first observed by Lachyankar et al. in nerve growth factor-treated PC12 cells and brain-derived neurotrophic factor-treated neurospheres (Lachyankar et al., 2000), and since that time a neuronal role for this component of PTEN has been sought. One function for nuclear PTEN is the regulation of the Anaphase-Promoting Complex (APC)/CDH1 complex through a protein-protein interaction yet independent of PTEN's phosphatase activity (Figure 1) (Song et al., 2011). Importantly, APC/Cdh1 has been previously shown to transcriptionally regulate genes associated with neuronal differentiation (De La Torre-Ubieta and Bonni, 2011). The nucleo-cytoplasmic shuttling of PTEN is regulated by the deubiquitinating enzyme HAUSP/USP7 (Figure 1), and cKO of HAUSP in the brain demonstrated profound phenotypes that may involve the loss of cytoplasmic PTEN (Kon et al., 2011). This possibility awaits further experiments as Kon et al. focused on the role of HAUSP regulation of p53. Their results indicated that p53 loss did not completely rescue the loss of HAUSP, leaving open a role for PTEN in HAUSP-cKO mice. A nuclear function for PTEN presents an intriguing link between the observed progenitor cell phenotypes in models of PTEN loss. This includes the observation that PTEN over-expression inhibits the induction of gene expression profiles consistent with neuronal differentiation in PC12 cells and that the effect is not due exclusively to perturbing growth factor signal, as PI3K and MAPK inhibitors were insufficient to replicate this effect (Musatov et al., 2004). This may indicate that excess PTEN protein may have sequestered crucial nuclear co-factors such as APC/CDH1 from their appropriate cellular locations and targets.

One of the mechanisms for PTEN nuclear import is through the action of the Nedd4 family-interacting protein 1 (Ndfip1) (Figure 1) (Hammond et al., 2013), a protein that has been shown to regulate PTEN in part through controlling ubiquitination, and by regulating PTEN secretion (Putz et al., 2012; Hopkins et al., 2013). This pool of extracellular and potentially trans-cellular PTEN offers attractive and yet-to-be-tested possibilities regarding non-cell autonomous roles for PTEN signaling during brain development. Interestingly, Ndfip1 is expressed in the developing brain, and conditional deletion of Ndfip1 in embryonic cerebral cortex using Emx1^Cre^ results in alterations of neuronal morphology, but does not impact neuronal number or specification (Goh et al., 2013; Hammond et al., 2013). While this work did not directly explore the impact of Ndfip1 loss on the subcellular distribution of PTEN, it did show that total PTEN levels appeared unchanged. However, studies of PTEN redistribution to the nucleus following ischemia find that Ndfip1-deficient animals do not import PTEN following anoxic injury, and that over-expression of Ndfip1 increased PTEN import into the nucleus (Howitt et al., 2012). Additional studies will be required to determine which of the observed phenotypes in the Ndfip1 null brain may be attributable to a lack of nuclear PTEN or perhaps other Ndfip1 targets. It is important to note that neither the nuclear nor cytoplasmic pool of PTEN alone can explain the observed deficits in the animal models we have discussed, but rather it is the concerted efforts of both pools of PTEN that orchestrate appropriate nervous system development.

## Cell migration

The appropriate localization and distribution of stem cell progeny make cell migration a key event in proper brain formation. Given the central role of PTEN in the transduction of many pathways that shape cellular responses to chemo-attractant or -repellent cues, it would be reasonable to expect that certain aspects of cell migration would be perturbed following the loss of PTEN. Many studies of the developing brain find little indication of massive alterations in migration, but lamination defects have been observed following Nestin-Cre deletion (Groszer et al., 2001). However, it is unclear if this observation reflects defects in migration or in progenitor proliferation.

Neuronal helix-loop-helix protein-1 (Nex1)^Cre^ (Table 1) deletion of PTEN only targets neurons that have left the cell cycle and become fated as neurons. Immunohistochemical staining for lamination markers such as Tbr1 (early born neurons) indicated a less compact cerebral cortex with some Tbr1^+^ cells near the marginal zone, and labeling of later born neurons using BrdU birthdating or Cux1 staining appeared to have normal placement, indicating no major defect in migration or positioning (Kazdoba et al., 2012). Somewhat surprisingly, these mice did display an increased expression of Reelin, a secreted regulator of migration, relative to controls. It is possible that the lack of effect on migration could be due to inefficient NEX1^Cre^-mediated deletion of PTEN in these cells. Additional analyses of cortical structure using Cre recombinases that target earlier steps in neurogenesis (Emx1^Cre^ and hGFAP-Cre) do find significant alterations in cortical organization and hippocampal radial glial persistence, respectively (Lehtinen et al., 2011; Wen et al., 2013).

As described above, PTEN-null cells migrating from the SVZ/SEZ along the RMS have been observed to fall into two subsets of cells—those that prematurely differentiate within the SVZ/SEZ, and those that continue to migrate to the OB (Zhu et al., 2012). Live imaging studies of this second group of cells indicated that their migratory speed was not statistically distinguishable from wild-type cells. The identity of the factors that distinguish these two classes of migrating cells may hold a wealth of information regarding chemotactic strategies employed by various RMS-transiting neuroblasts.

Early studies of conditional PTEN loss in the brain detected migration defects in the granule cells of the cerebellum (Backman et al., 2001; Kwon et al., 2001). Interestingly, while deletion of the Akt-activating kinase, Phosphoinositide-dependent kinase-1 (Pdk1), reverses some phenotypes observed in GFAP^Cre^/PTEN cKO mice (see **Somal Hypertrophy** below), it does not rescue cerebellar granule cell migration defects (Chalhoub et al., 2009). Experiments using L7/PCP2-cre to target PTEN deletion in Purkinje cells of the cerebellum did not detect a major alteration in cell placement, but ablation of PTEN by En2-Cre resulted in a significant alteration in organization involving essentially all constituent cells of the cerebellum (Marino et al., 2002). Later work using hGFAP-Cre and viral delivery of Cre suggested that the migration defect observed for granule cells is the result of premature differentiation of Bergmann glia rather than a cell autonomous effect (Yue et al., 2005). Taken together, these results highlight the intrinsic diversity related to cell migration cues, cellular responses and the multivalent ways in which PTEN may shape them.

## Cell polarity

PTEN plays a role in acquisition of polarity by augmenting signal transduction from extracellular cues. Much of the work exploring the role of PTEN in polarity and axon outgrowth has focused on its phosphatase activity during membrane receptor signaling. Experiments specifically exploring the ability of PTEN to alter axon specification place it as a key element of axogenesis. Over-expression of PTEN results in a failure of axon formation and neurite outgrowth (Shi et al., 2003; Jiang et al., 2005). Conversely, RNA interference (RNAi) blockade of PTEN expression leads to the formation of multiple axon projections (Jiang et al., 2005). *Caenorhabditis elegans* PTEN ortholog *daf-18* mutant neurons fail to properly polarize and extend axons (Adler et al., 2006), demonstrating the high degree of conservation across species. Additionally, *in vivo* loss-of-function studies using Neural Specific Enolase (NSE)-Cre to generate cKO PTEN mice indicate exuberant axon projections in the dentate gyrus (Kwon et al., 2006a). It will be important in future studies to assess whether these axonal defects are due to axon mis-targeting or altered neuronal polarity, but it is clear that axonal development is significantly altered following PTEN ablation in many types of neurons. While these studies make a compelling case for PTEN requirement during axon formation, these results do not delineate a specific role for PTEN in the early asymmetry events in axon specification or a role in regulation of axon outgrowth. Clarity for this morphologic aspect of PTEN function will await further investigation and new tools to probe the earliest events in neuronal polarization.

## Axon/dendrite outgrowth

Beyond affecting early neurite specification, PTEN loss has been associated with alterations in both axonal and dendritic structure. The nuclear pool of PTEN may contribute to this process as well through its interaction with APC/CDH1. This complex has been shown to regulate axon formation and outgrowth by targeting transcriptional components for degradation. As described earlier, PTEN could be contributing to the timing of axon specification/outgrowth by increasing the association of APC and CDH1 (Lasorella et al., 2006; Stegmuller et al., 2006). The related complex of APC/CDC20 has been implicated in axonal outgrowth (De La Torre-Ubieta and Bonni, 2011), but whether PTEN associates with this complex as well remains to be determined. PTEN/daf-18 has also been shown in multiple species to be crucial for shaping of neuronal morphology by regulating the transcription factor FoxO/daf-16 (Christensen et al., 2011) and by controlling the PI3K-Akt signaling pathway.

Axon projections were more exuberant in both dopamine transport promoter (DAT^Cre^) (Diaz-Ruiz et al., 2009; Domanskyi et al., 2011; Inoue et al., 2013) and Nse-Cre (Kwon et al., 2006a) deleted PTEN mouse lines. Adult newborn neurons respond to PTEN depletion much like embryonic cells, as retroviral siRNA *in vivo* increased axon diameter and bouton size (Luikart et al., 2011). However, midbrain dopaminergic neurons targeted for PTEN deletion using DAT^Cre^ did not display an increase in terminal bouton size (Diaz-Ruiz et al., 2009; Inoue et al., 2013). In contrast, PTEN deletion using Ca^2+^/Calmodulin-dependent protein kinase 2 promoter-driven (CaMKIIα)-Cre in mature neurons of the forebrain resulted in altered morphologies of neuronal processes (Sperow et al., 2012).

Work in Xenopus spinal neurons using pharmacologic inhibition of PTEN by Bisperoxo (1, 10-phenanthroline) oxovanadate, morpholino knock-down, or catalytically-inactive PTEN over-expression enhances axon growth when encountering target muscle tissue (Li and Peng, 2012). This suggests a role for PTEN in target recognition or down-regulation of axon growth signaling. Furthermore, spinal neuron growth cone chemotaxis has been shown to utilize PTEN selectively in response to chemorepulsive cues, demonstrating a potential context-dependent difference in PTEN modulated responses (Henle et al., 2013). Loss of the spinal motor neuron 1 gene (*SMN1*) leads to spinal motor atrophy and the axon outgrowth and growth cone defects associated with the loss of the *SMN1* gene expression can be rescued by siRNA knockdown of PTEN (Ning et al., 2010). As the SMN1 protein plays a role in both small nuclear ribonucleic particle regulation and axonal RNA transport, this result may indicate that PTEN contributes to the regulation of SMN1-regulated translation for axonal RNA through regulation of mTOR signaling, or that the local translation of PTEN itself may be increased following the loss of SMN1, or both. During axon growth, PTEN may be the target of mRNA stability/translational regulation via the microRNA cluster 17-92 member miR19 (Figure 1) (Zhang et al., 2013), as described above in the regulation of neural stem cells.

Experiments directed at understanding the ubiquitin proteasome system in neurons led to the finding that the ubiquitin ligase NEDD4 is capable of targeting PTEN for degradation (Figure 1) (Drinjakovic et al., 2010). This work also demonstrated that PTEN degradation is crucial for the regulation of axon branching. Recently, adult dorsal root ganglion axons were also shown to utilize NEDD4 to regulate PTEN during axon growth (Christie et al., 2012). This work highlights the significance of localized regulation of protein expression and signaling responsiveness of cellular compartments such as growth cones.

Similarly, dendrites are also affected by loss of PTEN. Most studies of PTEN deletion in the nervous system that detected changes in somatic size (see below) also report primary dendrites of enlarged caliber. A systematic study of the PI3K-Akt-mTOR signaling cascade in dendrite development found that shRNA reduction of PTEN resulted in increased branching consistent with increased activity in this transduction cascade (Jaworski et al., 2005).

## Somatic hypertrophy

The importance of the mTORC1 signaling pathway and its regulation by PTEN are apparent in the cellular hypertrophy that results from PTEN loss (Backman et al., 2001). This effect is reversed in neurons by inhibitors of mTOR kinase activity (Kwon et al., 2003; Ljungberg et al., 2009; Zhou et al., 2009). Similar hypertrophy is also observed in astrocytes (Fraser et al., 2004), but there is some indication that this phenotype may be sensitive to the timing of PTEN loss. Embryonic deletion in post-mitotic neurons using NEX1^Cre^ (Kazdoba et al., 2012) as well as DAT^Cre^ mice (Diaz-Ruiz et al., 2009; Domanskyi et al., 2011; Inoue et al., 2013) resulted in somatic hypertrophy, but deletion in mature, post-natal forebrain neurons using CaMKIIα-Cre did not have this effect (Sperow et al., 2012). Paralleling this result, virally-mediated shRNA knock-down of PTEN in adult born dentate gyrus neurons resulted in somatic hypertrophy, but this effect is delayed relative to neonatally injected animals (Luikart et al., 2011). Epistasis experiments pursuing the components of the pathway responsible for hypertrophy have shown that loss of the kinase Pdk1 leads to reversal of hypertrophy in GFAP^Cre^; PTEN^fl/fl^ mice (Chalhoub et al., 2009). Loss of S6K1, a key downstream effector of mTORC1 linking it to protein translation regulation, did not have the same effect (Chalhoub et al., 2006). The disruption of the regulated transport of PTEN in neurons by the motor protein Myosin V has also been shown to result in somatic hypertrophy (Figure 1) (Van Diepen et al., 2009). In this case, a direct interaction between these two proteins was shown to exist and to be regulated by either casein kinase 2 or glycogen synthase kinase 3 beta (GSK3β). These two studies demonstrate that a number of PTEN regulatory pathways can contribute to hypertrophy. An exact delineation of the mechanisms capable of influencing somatic hypertrophy await further studies to dissect the individual contributions of each PTEN regulator.

## PTEN and myelination

An important corollary to PTEN's effect on neuronal process outgrowth is the observation of myelination defects following PTEN loss (Fraser et al., 2008) and the hypermyelination by oligodendrocytes and Schwann cells lacking PTEN following deletion by oligodendrocyte transcription factor 2 (Olig2)^Cre^ (Harrington et al., 2010; Maire et al., 2014). 2', 3'-cyclic nucleotide 3' phosphodiesterase (CNP1)^Cre^ or Proteolipid protein 1 (Plp1) ERT2-Cre (Goebbels et al., 2010). Interestingly, rapamycin treatment of animals exhibiting altered myelination resulted in amelioration of the phenotype (Goebbels et al., 2010). This points to the lipid phosphatase activity of PTEN, and its antagonism of the PI3 kinase/mTOR pathway. A recent report has implicated PTEN as a key component of the polarized growth and wrapping of the myelin sheath at its inner tongue via regulation of the maturation state of the myelinating cell (Snaidero et al., 2014). Conditional deletion of PTEN using the Plp1-ERT2-Cre line resulted in a larger inner tongue and more cytoplasmic channels relative to controls, a sign of less mature myelinating sheets. Cytoplasmic-rich edges of the sheath in longitudinal sections that were absent in controls either 23 or 60 days post-natally were also seen (Goebbels et al., 2010; Snaidero et al., 2014). Deletion of PTEN at postnatal day 100 led to the reappearance of cytoplasmic myelin transport channels and increased myelination, relative to controls (Snaidero et al., 2014). These data indicate that it is possible to reinitiate the myelination program, even in mature animals, by increasing PI(3,4,5)P3 levels through loss of PTEN activity. This establishes PTEN both as a key regulator of embryonic development, and as a potential therapeutic target for myelination disorders and demyelinating diseases.

The profound effect on the inner tongue may be related to the localization of PTEN within the myelin sheet through interaction with scaffolding proteins such as Discs large homology 1 (Dlg1) or PAR-3. Reduction of Dlg1 has been shown to increase myelination and decrease levels of PTEN, suggesting a reciprocal stabilizing relationship between these two proteins (Cotter et al., 2010). However, neither Olig2^Cre^ nor CNP1^Cre^ PTEN cKO mice exhibit alterations in Dlg1 levels (Maire et al., 2014), (Goebbels et al., 2010), but there may be small, but significant, changes in signaling in these mice. Alternatively, the observed hypermyelination may reflect an alternate PTEN partner since myelination is also coordinated by a complex of the polarity protein PAR-3 and the neurotrophin receptor p75NTR (Chan et al., 2006). PTEN binds PAR-3 (Von Stein et al., 2005; Wu et al., 2007; Feng et al., 2008), and p75NTR receptor stimulation can increase PTEN activity (Song et al., 2010), thus it is possible that together as a tripartite complex these proteins could regulate axon ensheathment. However, much like Dlg1, localization of PAR-3 was not altered in CNP1^Cre^; PTEN^flox/flox^ mice (Goebbels et al., 2012). While the identity of the molecules targeting PTEN within myelinating cells may not be resolved, the important role for PTEN/PI3K/mTOR signaling is clear.

## Conclusions and future directions

Since its discovery almost 20 years ago, PTEN has remained a centerpiece of inquiry into signal transduction for a vast array of disciplines including tumor biology, metabolism, and neuroscience. Work over the last decade and a half has significantly strengthened our understanding of the major neurodevelopmental roles for PTEN. Going forward, a clearer understanding of the catalytic and non-catalytic functions of PTEN during brain development will be crucial, particularly in terms of transcriptional regulation. Similarly, the phosphatase activity linked to protein versus lipid dephosphorylation will expand our compendium of targets regulated by PTEN during nervous system patterning. Systematically perturbing PTEN interacting partners during brain development will allow us to begin to form a holistic view of how PTEN loss leads to the brain phenotypes observed.

It will also be important to develop “next-generation” tools with which to interrogate PTEN function in the developing brain. These might include chemical genetic variants of PTEN for rapid and specific elimination of phosphatase activity either in cell lines or mouse models, and inducible transgenic lines that permit cell-type specific rescue either with a doxycycline-regulated system or Cre-induced expression. To define functionality and understand the consequence of PTEN interacting with binding partners, point mutant variants should be developed that remove binding sites for particular partner proteins. Alternatively, systems can be devised that permit pharmacologic regulation of PTEN association with partner molecules. Forms of PTEN that are inducibly targeted to the sub-cellular locales known to be key sites of action for PTEN would also provide a mechanism to discern the various roles of PTEN within cellular compartments. Combinations of these and other future technologies will permit us to further delineate the PTEN-dependent mechanisms responsible for sculpting the developing and adult brain. Furthermore, the continued synergy with other disciplines that have already contributed significantly to our understanding of PTEN will continue to shape the field and provide new insights into PTEN biology enhancing our awareness of the role of PTEN in the brain.

### Conflict of interest statement

The authors declare that the research was conducted in the absence of any commercial or financial relationships that could be construed as a potential conflict of interest.
